# Taxonomic and functional diversity of microbial community from a mining environment

**DOI:** 10.1186/1471-2105-16-S8-A3

**Published:** 2015-04-30

**Authors:** Julliane D Medeiros, Laura R Leite, Sara Cuadros-Orellana, Guilherme Oliveira

**Affiliations:** 1 Universidade Federal de Minas Gerais, Minas Gerais, Brazil; 2 CPqRR/Fiocruz, Minas Gerais, Brazil

## Background

Biomining uses acidophilic and chemolithotrophic microbes capable of oxidizing iron and sulfur to recover metals of interest from complex minerals. One of the most critical issues in mining environments is the generation of acid mine drainage (AMD) that pollutes water and sediments with acids and metals. The Sulfate-reducing bacteria (SBR) are an alternative to bioremediate contamination of AMD. The current knowledge on the microbial diversity and the metabolic pathways involved in bioming and bioremediation is still limited. In this context, metagenomics has become a valuable tool to investigate previously uncultured microorganisms in environmental samples. The aim of this study was to assess the taxonomic and functional microbial diversity in a mining area in the Brazilian Amazon.

## Results

We collected acid mining drainage water; sediment and water from the surface and 15 meters depth of a tailings dam that received ~90 million tons of chalcopyrite mining waste. The prokaryotic biomass from water samples was concentrated on filters with 0.22m pores. Metagenomic DNA was isolated and the V4 region of the 16S rRNA was amplified. Shotgun and amplicon libraries were sequenced on Ion Torrent platform. We used Qiime to cluster the sequences into OTU and we observed that the microbial diversity was higher on the sedi- ments and AMD metagenomes (Sediment - Chao:1988; Shannon:6.05 / AMD - Chao:2375; Shannon:5.03) compared to the water (Chao:370; Shannon:4.16). MG-RAST was used to classify the reads (representative hit, identity cut-off >=75%) Biomining uses acidophilic and chemolithotrophic microbes capable of oxidizing iron and sulfur to recover metals of interest from complex minerals. We collected acid mining drainage water; sediment and water from the surface and 15 meters depth of a tailings dam that received ~90 million tons of chalcopyrite mining waste. The prokaryotic biomass from water samples was concentrated on filters with 0.22 >=75%), and the results indicate the dominant phyla on water metagenomes were Proteobacteria (49.3%), Actinobacteria (31.6%) and Bacteroidetes (7.6%). The most abundant phyla in sediments were Proteobacteria (48.7%) Bacteroidetes (17.2%) and Firmicutes(7.5%). We analyzed OTUs from specific SRB-families (Thermodesulfovibrionaceae, Desulfobacteraceae, Syntrophaceae, Peptococcaceae, Desulfuromonadaceae, Desulfomicrobiaceae) and there was an enrichment of these families in sediment and AMD samples. Blastx against NR was used for functionally classify reads in MEGAN using the SEED and KEGG hierarchy.

## Conclusions

Despite the prevalence of housekeeping functions, we observed reads matching relevant adaptive traits, such as sulfur oxidation, ABC transporters and resistance to metal. Our data indicate the potential for biomining and biore- mediation on the studied environments, however new rounds of sequencing must be done for more conclusive results.

